# Toward an algorithm of percutaneous microelectrolysis: a randomized clinical trial on invasive techniques

**DOI:** 10.1590/1516-3180.2024.0164.R1.07032025

**Published:** 2025-08-11

**Authors:** Carlos Eduardo Girasol, Nathaly Escobar Durán, Santiago Marcelo D’Almeida, Oscar Ariel Ronzio

**Affiliations:** I Postdoctoral Researcher, and Clinical Physiotherapist, Faculdade de Filosofia, Ciências e Letras, Universidade de São Paulo (USP), Ribeirão Preto (SP), Brazil.; II Researcher, and Clinical Physiotherapist, Cuauhtémoc Plantel Aguascalientes University, Mérida, Mexico.; III Professor, Researcher, and Clinical Physiotherapist, Maimónides University (UMai), Buenos Aires, Argentina.; IV Professor, Researcher, and Clinical Physiotherapist, Arturo Jauretche Nacional University (UNAJ), Florencio Varela, Argentina.

**Keywords:** Electrolysis, Electric stimulation therapy, Physical therapy modalities, Galvanic current, Myofascial pain syndromes, Pain management

## Abstract

**BACKGROUND:**

Percutaneous microelectrolysis (MEP) is a minimally invasive technique used for pain relief, inflammation control, and tissue repair. However, the optimal treatment protocol remains under debate.

**OBJECTIVE:**

To compare the effects of dry needling and MEP, with and without a treatment algorithm, on pain in individuals with active myofascial trigger points (MTrPs) in the upper trapezius muscle. Design and setting: Randomized controlled trial conducted at Maimónides University, Buenos Aires.

**METHODS:**

Eighty-eight participants with MTrPs in the upper trapezius muscle were enrolled. The presence of MTrPs was confirmed through physical examination and algometric measurement before intervention. Participants were randomly assigned to one of six groups: Sham, dynamic dry needling, static dry needling, dynamic MEP, static MEP, or algorithmic MEP. Active treatments were administered using 0.30 mm × 40 mm acupuncture needles. Pain was assessed using two tools: the Numerical Pain Rating Scale (NPRS) and the Pressure Pain Threshold (PPT). Both measures were recorded with participants at rest before the intervention and again at 10 min, 24 h, 48 h, and 7 days post-intervention.

**RESULTS:**

Significant post-intervention differences in NPRS scores were observed in all groups except dynamic dry needling when compared to Sham. The algorithmic MEP group achieved complete pain relief by day 7. In terms of PPT, the threshold values in the MEP groups were lower than those in the other groups.

**CONCLUSIONS:**

All needling techniques demonstrated analgesic effects on myofascial trigger points, with the algorithm-enhanced MEP showing the most notable improvement in self-reported pain. However, MEP was not superior to other methods in improving pressure pain thresholds.

**CLINICAL TRIALS:**

NCT05478928.

## INTRODUCTION

 Myofascial pain syndrome is a non-inflammatory disorder characterized by localized pain and stiffness, with the hallmark feature being the presence of myofascial trigger points (MTrPs).^1^ While several diagnostic criteria exist, the primary indicator is muscle irritability triggered by pressure or stretching, which produces localized tenderness or referred pain. A palpable taut band may be detected, representing a localized muscle spasm.^1,2^ The associated pain is typically constant and deep, and myofascial pain is considered a highly prevalent condition, often serving as a common reason for individuals to seek medical care.^3^

 Several treatment modalities are available for this syndrome, including electrophysical agents.^4-6^ One of the oldest such modalities is direct current (DC), also known as galvanic current, which is defined by its unidirectional flow. The energy accumulated in stimulated biological tissues during DC application promotes electrochemical changes, referred to as polar effects.^7^ More recently, the use of DC in invasive applications has gained prominence in rehabilitation,^8,9^ particularly through techniques such as percutaneous microelectrolysis (MEP).^10^ MEP is a minimally invasive procedure in which low-intensity DC (up to 0.98 mA) is delivered via acupuncture needles, generating a high current density (approximately 3.8 mA/cm^2^).^11^ In addition to providing local analgesia, MEP is thought to induce a controlled inflammatory response that facilitates tissue repair. These physiological effects support its application in treating MTrPs, which are commonly found in the trapezius muscle among individuals with neck pain.^12^

 However, the optimal intervention protocol for this therapy remains under debate, particularly regarding patient comfort during treatment. To address this, an algorithm has been proposed to minimize discomfort. Therefore, the aim of this study was to compare the effects of dry needling and percutaneous microelectrolysis, with or without the proposed algorithm, on pain in individuals with MTrPs in the upper trapezius muscle. 

## METHODS

### Study design

 This single-blind, randomized controlled trial was conducted using a convenience sample that included participants of both sexes, aged 18–48 years, with active MTrP, either unilateral or bilateral. Researcher 1, who was responsible for the statistical analysis, and the participants were blinded to group assignments. Recruitment, randomization, and group allocation were carried out by Researcher 2, who was not involved in participant assessment or intervention. Blocked randomization and blinded allocation were performed using the website: www.randomizer.org. Allocation concealment was ensured through sealed opaque envelopes, which were opened only by Researcher 3, responsible for administering the intervention, after the evaluation and immediately before the intervention. Both the participants and Researcher 3 remained unaware of the specific intervention until the treatment protocol was initiated. As a result, only the researcher performing the statistical analysis remained fully blinded throughout the study. 

### Ethical aspects

 The study was approved by the Ethics Committee of Maimónides University (A-01-CEBBAD-20), in accordance with the Declaration of Helsinki and national ethical standards. The trial was registered on ClinicalTrials.gov (NCT05478928). All participants were fully informed about the nature, objectives, and procedures of the study, and each provided written informed consent. The study followed all guidelines outlined by the CONSORT 2010 (Consolidated Standards of Reporting Trials) statement.^13^

### Participants

 The criteria established by Simons et al.^1^ and Gerwin et al.^14^ were used to define MTrP: the presence of a taut band in the skeletal muscle, a hypersensitive spot within the taut band, a local contraction response upon palpation, and reproduction of referred pain when applying up to 3 kg/cm^2^ of pressure on the trigger point.^15^ These diagnostic criteria have demonstrated acceptable reliability, with κ values ranging from 0.36 to 0.882.^14^ Participants were excluded if they had a history of neck or shoulder surgery, needle phobia, temporomandibular dysfunction, use of anticoagulants, concurrent treatment for MTrPs, fibromyalgia, endocrine disorders, pregnancy, or obesity (defined as a body mass index [BMI] greater than 28 kg/m^2^).^16^

### Assessment procedures

 Assessments were conducted before the first treatment session and at 10 min, 24 h, 48 h, and 7 days following the intervention. The following outcomes were evaluated: the Numerical Pain Rating Scale (NPRS) and the Pressure Pain Threshold (PPT). The primary outcomes of the study were pain intensity and pressure pain threshold at the MTrP 

### Anamnesis and physical examination

 During the anamnesis, the examiner collected demographic and clinical information, including personal data, weight (kg), height (m), BMI (kg/m^2^), occupation, history of illness, medication use, and any history of surgery or physical therapy. 

 Pain intensity was measured using the NPRS, a simple and validated tool consisting of a scale from 0 to 10, where 0 represents "no pain" and 10 represents "the worst pain imaginable."^17^

 The PPT was measured using an FDX® 25 algometer (Wagner Instruments, Greenwich, United States). A trained examiner placed the 1 cm^2^ tip of the algometer perpendicular to the upper trapezius muscle fibers, bilaterally, just above the MTrP. Pressure was applied gradually at a rate of approximately 0.5 kg/cm^2^/s. This instrument has excellent intra- and inter-rater reliability (0.752 and 0.874, respectively).^18^ Each site was compressed three times until the participant reported the onset of pain, and the average of the three readings (in kg/cm^2^) was recorded. Both PPT and NPRS assessments were performed while the participant was at rest, before the intervention, and again at 10 min, 24 h, 48 h, and 7 days post-intervention. 

### Intervention

 Participants were randomly assigned to one of six groups: Sham, dynamic dry needling, static dry needling, dynamic MEP, static MEP, or algorithmic MEP. 

 In the dry needling groups, a single needle was inserted into the MTrP and positioned perpendicular to the upper trapezius trigger point for 120 s. In the static dry needling group, the needle remained stationary throughout the duration. In the dynamic dry needling group, needle movements were performed at a frequency of 1 Hz for the full 120 s. 

 In the MEP groups, a Sveltia DC device was used, connected to a dispersive electrode (area: 28.26 cm^2^) and an acupuncture needle (0.3 × 2.5 cm). The circuit was completed by placing the dispersive electrode on the arm contralateral to the trapezius muscle being treated. The current intensity at the needle was 0.6 mA, resulting in a current density of 2.53 mA/cm^2^. 

 In the static MEP group, the needle was kept stationary for 120 s during active stimulation, delivering a total dose of 72 mC. In the dynamic MEP group, needle movements at 1 Hz were performed throughout the same period. If participants reported discomfort during any MEP intervention, the procedure was paused and resumed only once the discomfort subsided. 

 In the algorithmic MEP group, the needle was held stationary, but the treatment continued until the participant reported no discomfort for more than 60 s. No predefined time limit exists for signal cessation. In the Sham group, a 0.3 × 25 mm acupuncture needle was inserted perpendicularly to a depth of 3 mm into the upper trapezius trigger point and held without movement for 120 s. 

**Figure 1:**
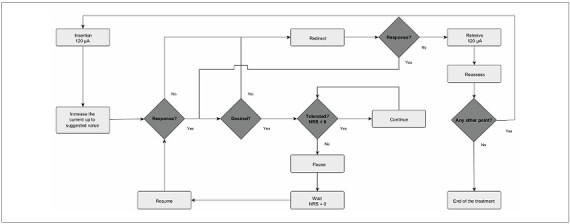
Proposed algorithm for the execution of percutaneous microelectrolysis.

 A detailed description of the algorithm is provided in Figure 1, and the intervention parameters for all interventions are summarized in Table 1. 

**Table 1:** Parameters proposed for intervention

**Parameters**	**DN Dynamic**	**MEP Dynamic**	**DN Static**	**MEP Static**	**MEP Static Alg.**	**Sham**
Needle	0.3 × 2.5 cm	0.3 × 2.5 cm	0.3 × 2.5 cm	0.3 × 2.5 cm	0.3 × 2.5 cm	0.3 × 2.5 cm
Dispersive Electrode Area	-	28.26 cm^2^	-	28.26 cm^2^	28.26 cm^2^	-
Frequency of movements	1 Hz	1 Hz	-	-	-	-
Current Intensity	-	0.6 mA	-	0.6 mA	0.6 mA	-
Time	120 s	120 s	120 s	120 s	variable	120 s
Total Dose	-	72 mC	-	72 mC	variable	-
Intervention Place	Perpendicular to the trigger point of the upper trapezius muscle					-

DN: Dry needling; MEP: Microelectrólisis Percutaneous; Alg.: Algorithm

**Figure 2:**
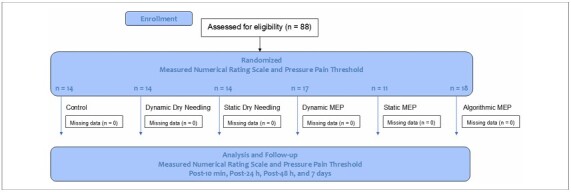
Research process flowchart.

 The flowchart of the study development is shown in Figure 2. 

### Statistical analysis

 Statistical analysis was performed using IBM SPSS Statistics for Windows (version 20.0, IBM Corp., Armonk, New York). Descriptive statistics were used to report the means and standard deviations of participant characteristics. The normality of the data was assessed using the Shapiro–Wilk test. A two-way repeated measures analysis of variance (ANOVA) was conducted to evaluate the interaction between group and time. Results were reported as mean differences with 95% confidence intervals (95% CI) for the outcomes NPRS and PPT. Tukey’s post hoc test was used for multiple comparisons, with the significance level set at P = 0.05. Data were reported as mean differences with corresponding standard errors. Effect sizes were calculated using Cohen’s d, and interpreted according to Cohen’s classification:^19^ small (< 0.2), moderate (near 0.5), and large (> 0.8). 

## RESULTS

### Compliance with the study protocol

 The study initially planned to use the Visual Analog Scale; however, during implementation, the NPRS was adopted to improve participant understanding and streamline data collection. All participants completed their assigned intervention session. No participants reported receiving additional treatments during the study, and no adverse events were noted following any of the interventions. 

### Flow of participants through the study

 A total of 88 participants (55 females) were recruited. The flow of participants and any loss to follow-up are illustrated in Figure 2. 

### Characteristics of the participants

 Descriptive statistics for each intervention group are provided in Table 2. The six groups were well balanced at baseline. Most participants (62.5%) were female, and most were young adults. 

**Table 2:** Mean (SD; CI 95%) of demographic and clinical characteristics of participants

**Outcomes**	**DN Dynamic**	**MEP Dynamic**	**DN Static**	**MEP Static**	**MEP Static Alg.**	**Sham**
n	14	17	14	11	18	14
Sex (Female %)	9 (64.3)	13 (76.5)	8 (57.1)	9 (81.8)	7 (38.9)	9 (64.3)
Age (years)	37.1 (8.45;32.8–42.5)	20.9 (2.37;19.3–21.8)	34.9 (9.77;26.4–39.8)	18.7 (0.79;18.2–19.3)	20.2 (1.79;19–21.6)	28.9 (8.32;23.4–35.2)
Weight (kg)	79.1 (13.1;70.5–88.8)	69 (12.6;58.7–73)	79.3 (20.9;68.2–86.3)	61.5 (8.85;55.6–67.5)	64.4 (9.02;55.7–69.1)	75.4 (17.5;65.3–90.3)
Height (m)	1.69 (0.09;1.6–1.8)	1.67 (0.1;1.6–1.7)	1.7 (0.1;1.7–1.8)	1.65 (0.08;1.6–1.7)	1.71 (0.06;1.6–1.7)	1.73 (0.1;1.7–1.8)
BMI (kg/m^2^)	27.7 (4.14;24.8–30.9)	24.8 (3.5;21.7–26.3)	27 (5.04;24–28.5)	22.5 (1.75;21.3–23.7)	22 (2.17;20.1–23.5)	25 (4.13;22.1–28.2)
PPT Pre	2.3 (0.58;1.8–2.7)	1.81 (0.58;1.3–2.1)	2.40 (0.37;2.1–2.6)	1.95 (0.65;1.4–2.4)	1.74 (0.54;1.2–1.9)	2.40 (0.25;2.4–2.3)
PPT Post-10 min	2.66 (0.36;2.5–2.8)	1.39 (0.54;1–1.8)	2.45 (0.3;2.3–2.7)	1.28 (0.67;0.7–1.7)	1.54 (0.61;1.1–1.8)	2.54 (0.35;2.2–2.8)
PPT Post-24 h	3.82 (1.98;2.4–5.6)	1.29 (0.47;0.9–1.5)	3.08 (0.75;2.4–3.6)	1.39 (0.52;1–1.8)	1.65 (0.45;1.4–2)	3.35 (1.5;2.2–4.8)
PPT Post-48 h	3.96 (2;2.4–5.7)	1.86 (0.60;1.4–2.1)	3.53 (0.98;2.6–3.9)	1.6 (0.69;1.1–2.1)	1.78 (0.56;1.4–2.2)	3.67 (1.42;2.7–5)
PPT Post-7 days	4.09 (2.21;2.4–6)	2.08 (0.93;1.4–2.5)	3.81 (0.93;2.9–4.2)	1.77 (0.75;1.2–2.3)	1.96 (0.65;1.6–2.2)	3.74 (1.51;2.7–5.2)
NPRS Pre	6.5 (1.79;5.5–8.1)	6.12 (2.12;3.6–6.8)	6.29 (2.09;4.7–7.5)	5.55 (1.37;4.5–6.5)	5.06 (1.34;3.7–5.9)	5.93 (2.59;4–7.8)
NPRS Post-10 min	6.93 (1.73;5.1–7.5)	3.18 (2.35;0.8–4.8)	5.5 (1.4;4.6–6.2)	2 (2.65;−0.1–3.9)	2.38 (1.93;0.6–3.2)	5.71 (2.3;3.7–7.3)
NPRS Post-24 h	4.29 (3.24;1.4–6.4)	3.29 (2.39;1.3–5.3)	4.29 (2.67;2.5–6.1)	1.09 (1.3;0.3–2.1)	0.94 (1.34;−0.2–1.8)	4.14 (3.63;1.3–6.9)
NPRS Post-48 h	3.57 (3.41;0.8–6.4)	0.88 (1.05;0.2–1.6)	3.71 (2.67;2.1–5.9)	0.27 (0.46;0–0.6)	0.31 (0.87;−0.4–1)	2.71 (3;0.6–4.6)
NPRS Post-7 days	2.5 (3.3;−0.1–5.1)	0.31 (0.79;−0.1–0.5)	2.64 (2.53;1.3–5.1)	0.45 (1.04;−0.3–1.3)	0 (0;0)	2.64 (3.13;0.3–4.5)

DN: Dry needling; MEP: Microelectrólisis Percutaneous; Alg.: Algorithm; BMI: Body Mass Index; PPT: Pressure Pain Threshold; NPRS: Numeric Pain Rating Scale; Values are reported as mean (SD)

### Effects of the interventions

 In group comparisons for the NPRS, significant post-intervention differences were observed across all groups, except for the dynamic dry needling group when compared to Sham. The Algorithmic MEP group was the only group to reach an NPRS score of 0 out of 10 on day 7, indicating complete resolution of pain. Regarding PPT, the MEP groups (Static, Dynamic, and Algorithmic) demonstrated lower thresholds compared to the other groups. All relevant data, along with the results of the two-way repeated measures ANOVA and corresponding effect sizes, are provided in Tables 3 and 4. Overall, the groups treated with percutaneous microelectrolysis showed superior outcomes on the NPRS. However, the dry needling groups exhibited better results for PPT. The Sham group consistently showed the least favorable clinical responses. 

**Table 3:** Outcomes of the interactions between time and group variables for Numerical Pain Rating Scale (NPRS) and Pressure Pain Threshold (PPT)

**Pressure Pain Threshold**							
**Moment**	**Comparison**	**Mean Diff.**	**Std. Error**	**P value**	**95% CI for difference**		**d_cohen_**
					**Lower**	**Upper**	
**Pre**	Sham vs. MEP Static Alg.	0.838	0.173	0.014	0.155	1.521	-
	DN Static vs. MEP Static Alg.	0.800	0.163	0.013	0.154	1.446	-
**Post-10 min**	Sham vs. MEP Dynamic	1.125	0.254	0.025	0.122	2.128	−1.18
	Sham vs. MEP Static	1.288	0.256	0.011	0.275	2.301	−0.277
	Sham vs. MEP Static Alg.	1.041	0.247	0.034	0.065	2.017	−0.756
	DN Dynamic vs. MEP Dynamic	1.265	0.243	0.008	0.303	2.227	1,31
	DN Dynamic vs. MEP Static	1.428	0.213	0.001	0.587	2.269	1.629
	DN Dynamic vs. MEP Static Alg.	1.181	0.188	0.002	0.438	1.924	0.979
	MEP Dynamic vs. DN Static	−1.078	0.234	0.019	−2.003	−0.153	−0.921
	DN Static vs. MEP Static	1.241	0.222	0.005	0.361	2.121	1.363
	DN Static vs. MEP Static Alg.	0.994	0.207	0.014	0.177	1.811	0.514
**Post-24 h**	DN Hong vs. MEP Dynamic	2.787	0.600	0.018	0.413	5.161	−3.425
	MEP Dynamic vs. DN Static	−1.801	0.241	0.001	−2.752	−0.850	2.352
	DN Static vs. MEP Static	1.609	0.194	0	0.830	2.388	−2.347
	DN Static vs. MEP Static Alg.	1.274	0.321	0.049	0.005	2.543	−1.584
**Post-48 h**	Sham vs. MEP Static	2.227	0.499	0.024	0.253	4.201	−3.347
	Sham vs. MEP Static Alg.	2.038	0.512	0.048	0.013	4.063	−2.734
	MEP Dynamic vs. DN Static	−1.486	0.326	0.021	−2.776	−0.196	−2.117
	DN Static vs MEP Static	1.613	0.343	0.017	0.257	2.969	−3.102
	DN Static vs. MEP Static Alg.	1.424	0.324	0.026	0.141	2.707	−2.242
**Post 7 days**	DN Static vs. MEP Static	1.786	0.253	0.001	0.785	2.787	−3.009
	DN Static vs. MEP Static Alg.	1.599	0.344	0.018	0.240	2.958	−2.448

**Table 4:** Numeric pain rating scale

**Moment**	**Comparison**	**Mean Diff.**	**Std. Error**	**P value**	**95% CI for difference**		**d_cohen_**
					**Lower**	**Upper**	
**Post-10 min**	Sham vs. MEP Static Alg.	3.600	0.763	0.016	0.583	6.617	−1.21
	DN Dynamic vs. MEP Static Alg.	4.400	0.792	0.005	1.270	7.530	−1.954
	DN Static vs. MEP Static	3.500	0.792	0.025	0.367	6.633	−1.473
	DN Static vs. MEP Static Alg.	3.500	0.719	0.013	0.658	6.342	−1.08
**Post-24 h**	DN Static vs. MEP Static	3.100	0.706	0.026	0.307	5.893	−1.313
	DN Static vs. MEP Static Alg.	3.500	0.860	0.042	0.101	6.899	−1.211
**Post-48 h**	MEP Dynamic vs. DN Static	3.100	0.767	0.044	0.068	6.132	1.23
	DN Static vs. MEP Static	3.700	0.817	0.021	0.469	6.931	−1.441
	DN Static vs. MEP Static Alg.	3.700	0.857	0.029	0.311	7.089	−1.24

## DISCUSSION

 Several studies have examined the effects of MEP in the context of rehabilitation.^20-22^ In the current study, in addition to evaluating clinical outcomes, we investigated the impact of incorporating an algorithm to guide a more targeted and patient-responsive treatment approach. The results demonstrated significant differences in both the NPRS and the PPT. 

 As discussed by D’Almeida et al.,^11^ the MEP is considered a safe and effective therapeutic modality, a view supported by Ortiz et al.^7^ and Ronzio et al.^10^ Moreover, other studies have explored the analgesic effects and PPT outcomes of various MEP applications. For example, Al-Boloushi et al.^23^ studied patients with plantar heel pain and found positive results in pain reduction, consistent with our findings. Similarly, favorable outcomes have been reported in conditions involving MTrPs, such as patellofemoral pain syndrome^21^ and temporomandibular myofascial pain,^20^ further supporting the clinical utility of percutaneous electrolysis. The observed benefits may be attributed to the mechanical effects of needle insertion and the chemical effects induced by the galvanic current, as previously described. 

 However, unlike other studies,^7^ our investigation did not find significant improvements in PPT among the MEP groups. We hypothesize that the inflammatory response triggered by DC may have sensitized the treated area, leading to reduced pressure tolerance and, consequently, lower PPT values.^10^

 An important point of discussion is that various forms of therapeutic electrolysis are currently under investigation, all of which are based on their ability to induce a localized and controlled inflammatory response within the treatment area. This response is consistently highlighted as a superior mechanism for modulating collagen synthesis and enhancing local circulation, thereby promoting tissue repair and pain relief. However, a key distinguishing factor among these techniques is the intensity of the electrical current used. The model employed in this study utilized microampere (μA) intensity, which is known to provide greater comfort during the intervention. Furthermore, the current findings align with the observations of Ortiz et al.,^24^ who emphasize the need for clearer standardization of treatment doses and protocols. In this context, the algorithm used in our study supports a more targeted and effective clinical approach based on current evidence. 

 All intervention groups showed analgesic effects, but only the algorithm group reached a pain score of zero by day 7. Including this group in future studies is strongly recommended, along with a larger sample size to validate these findings. The analgesic effects observed align with those reported in a recent systematic review.^25^ Therefore, the use of such an algorithm appears to be a valuable complement to invasive electrotherapy techniques. Nevertheless, this study has limitations, including a relatively small sample size and a lack of control for hormonal phase variations among female participants,^26,27^ which should be considered when interpreting the results. 

### Clinical relevance

- Needling techniques demonstrate analgesic effects on myofascial trigger points.
- The use of the proposed algorithm in conjunction with percutaneous microelectrolysis enhanced the analgesic effects.
- With regard to pressure pain threshold, the outcomes for the three percutaneous microelectrolysis techniques (Static, Dynamic, and Algorithmic) were comparable.

## CONCLUSION

 Needling techniques produce analgesic effects on myofascial trigger points, particularly when combined with the proposed algorithm. However, in terms of pressure pain threshold, the addition of microelectrolysis did not demonstrate superior results. 
